# Transcriptome Analysis of Rice Root Tips Reveals Auxin, Gibberellin and Ethylene Signaling Underlying Nutritropism

**DOI:** 10.1093/pcp/pcae003

**Published:** 2024-01-16

**Authors:** Kiyoshi Yamazaki, Yoshihiro Ohmori, Hirokazu Takahashi, Atsushi Toyoda, Yutaka Sato, Mikio Nakazono, Toru Fujiwara

**Affiliations:** Department of Applied Biological Chemistry, Graduate School of Agricultural and Life Sciences, The University of Tokyo, Yayoi, Bunkyo-ku, Tokyo, 113-8657 Japan; Department of Applied Biological Chemistry, Graduate School of Agricultural and Life Sciences, The University of Tokyo, Yayoi, Bunkyo-ku, Tokyo, 113-8657 Japan; Graduate School of Bioagricultural Sciences, Nagoya University, Furo-cho, Chikusa, Nagoya, 464-8601 Japan; Advanced Genomics Center, National Institute of Genetics, Mishima, Shizuoka, 411-8540 Japan; Department of Genomics and Evolutionary Biology, National Institute of Genetics, Mishima, Shizuoka, 411-8540 Japan; Graduate School of Bioagricultural Sciences, Nagoya University, Furo-cho, Chikusa, Nagoya, 464-8601 Japan; Department of Applied Biological Chemistry, Graduate School of Agricultural and Life Sciences, The University of Tokyo, Yayoi, Bunkyo-ku, Tokyo, 113-8657 Japan

**Keywords:** Differential cell growth, Laser microdissection, Nutrient, Plant root, Signal transduction, Tropism

## Abstract

Nutritropism is a positive tropism toward nutrients in plant roots. An NH_4_^+^ gradient is a nutritropic stimulus in rice (*Oryza sativa* L.). When rice roots are exposed to an NH_4_^+^ gradient generated around nutrient sources, root tips bend toward and coil around the sources. The molecular mechanisms are largely unknown. Here, we analyzed the transcriptomes of the inside and outside of bending root tips exhibiting nutritropism to reveal nutritropic signal transduction. Tissues facing the nutrient sources (inside) and away (outside) were separately collected by laser microdissection. Principal component analysis revealed distinct transcriptome patterns between the two tissues. Annotations of 153 differentially expressed genes implied that auxin, gibberellin and ethylene signaling were activated differentially between the sides of the root tips under nutritropism. Exogenous application of transport and/or biosynthesis inhibitors of these phytohormones largely inhibited the nutritropism. Thus, signaling and *de novo* biosynthesis of the three phytohormones are necessary for nutritropism. Expression patterns of *IAA* genes implied that auxins accumulated more in the inside tissues, meaning that ammonium stimulus is transduced to auxin signaling in nutritropism similar to gravity stimulus in gravitropism. *SAUR* and *expansin* genes, which are known to control cell wall modification and to promote cell elongation in shoot gravitropism, were highly expressed in the inside tissues rather than the outside tissues, and our transcriptome data are unexplainable for differential elongation in root nutritropism.

## Introduction

Tropisms enable plants to change their growth direction toward or away from external stimuli (Darwin 1897, Gilroy 2008). Numerous reports published over a century cover phototropism, gravitropism, hydrotropism and their underlying molecular mechanisms (Liscum et al. 2014, Muthert et al. 2020). Generally, physiological responses of tropisms are divided into three steps: perception of external stimuli, signal transduction and differential cell growth (elongation or division). While molecular mechanisms of perception under phototropism, gravitropism and hydrotropism depend on the stimulating signal, the signals are likely transduced to changes in phytohormone distributions (Muthert et al. 2020). Well-established signal transduction models of both root and shoot tropisms show the asymmetric distribution of auxins, which generate an asymmetric downstream cascade. For example, the PIN-FORMED (PIN) auxin transporter proteins change their polar localization depending on the directions of light and gravity, resulting in the generation of an asymmetric auxin distribution (Han et al. 2021). PIN polar localization in gravitropism is also regulated by other phytohormones, such as gibberellins (Lofke et al. 2013) and brassinosteroids (Li et al. 2005). Ethylene also affects auxin distribution in gravitropism (Muday et al. 2012). An asymmetric distribution of cytokinins causes root hydrotropism (Chang et al. 2019). Phytohormones enable plant cells to change the expression of various genes via regulation of their downstream transcription factors, resulting in differential growth as tropic responses. Auxin response factors (ARFs) and AUXIN/INDOLE-3-ACETIC ACIDs (AUX/IAAs) are key transcriptional regulators of auxin responses (Muday et al. 2012). ARF7 and ARF19 mediate transcriptional responses in phototropism and gravitropism in *Arabidopsis thaliana* and *Brassica oleracea* (Wang et al. 2020). Upregulation of the expressions of genes downstream of ARF7 and ARF19, such as *expansin* and *small auxin up RNA* (*SAUR*) genes, is required for differential cell elongation (Esmon et al. 2006, Wang et al. 2020). Cell walls of outside tissues of bending shoots must be softened through enzymatic modification for differential cell elongation to occur in phototropism (Liscum et al. 2014). One of the most studied enzymes for cell wall modification in phototropism and gravitropism is expansin. Enzymatic activity of expansin is enhanced under low apoplastic pH, and SAURs, present downstream of auxin signaling, promote apoplastic acidification in outside elongating tissues (Sampedro and Cosgrove 2005, Esmon et al. 2006, Majda and Robert 2018, Wang et al. 2020). Thus, asymmetric regulation of gene expression in response to an asymmetric distribution of phytohormones is required for known tropic responses. Comparison of transcriptome patterns between inside and outside cells would be an efficient way to reveal the genes regulated in the process of tropisms. In practice, RNA-seq analysis of upper (inside) and lower (outside) tissues from the bending hypocotyls indeed identified some gene sets contributing to gravitropism (Wang et al. 2020).

Recently, we discovered nutritropism in both lateral and main roots of rice (Yamazaki et al. 2020, Yamazaki and Fujiwara 2022). Rice roots exposed to an ammonium gradient generated around an ammonium source in agar medium showed nutritropism toward the source. Root tips show differential cell elongation under nutritropic bending (Yamazaki et al. 2020). The molecular mechanism, including gene expression profiles, remains unclear, and there are not even any reports about whether phytohormones are involved in signal transduction in nutritropism. Transcriptome analysis could reveal the genetic responses and phytohormone signaling required for nutritropism.

Here, we conducted laser microdissection (LMD) to separate root tips into inside and outside tissues. To distinguish the tissues, we developed a sampling method to retain spatial information on the directions of nutritropic stimuli in the root tips. Distinct transcriptome profiles between inside and outside tissues revealed the involvement of phytohormone signaling and cell wall modification. By the local application of inhibitors of phytohormone biosynthesis, we demonstrated that *de novo* biosynthesis of auxins, gibberellins and ethylene in root tips is essential for nutritropism.

## Results

### Gene expression data of inside and outside tissues in root tips during nutritropism

**Fig. 1:**
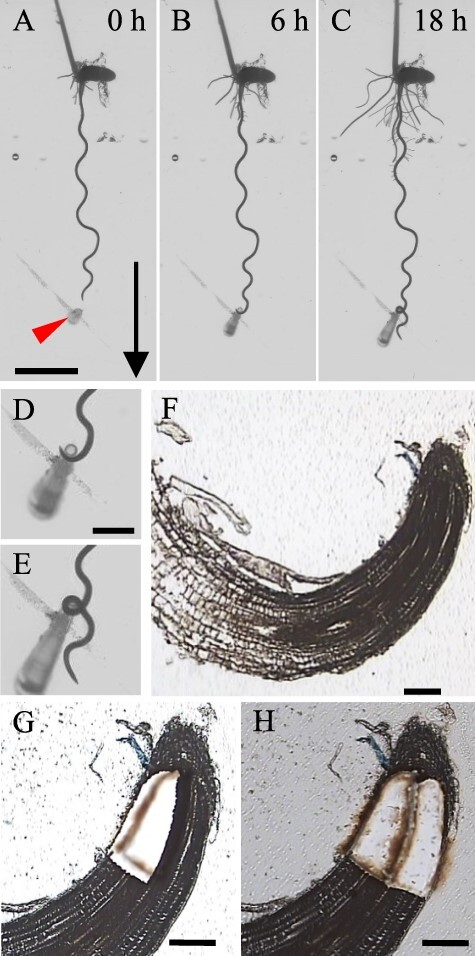
Laser microdissection for sample collection for RNA-seq. (A–C) Nutritropism in a main root of WRC25. (B, D) The root tip kept bending to coil around the nutrient source containing nutritropic stimulant (arrowhead) at ∼6 h. (C, E) After coiling, the root grew downward again. D and E are enlarged images of B and C, respectively. (F) Paraffin section of the coiling root tip. (G, H) From this section, cells were collected as inside and outside tissues. Arrow, gravitational direction. Scale bars: A–C, 10 mm; D, E, 2.5 mm; F–H, 100 μm.

Coiling roots appeared within 20 h after insertion of the nutrient sources (**Fig. 1A****–****E**). The root tips bent toward and coiled around the nutrient sources over several hours. The coiling was due to continuous nutritropic bending toward the source. We sampled the root tips at 12–24 h after the nutrient source insertion while the roots were coiling to capture the process of the nutritropic response. From the coiled shape in the section images (**Fig. 1F**), spatial information on the stimulus direction and the tropic bending direction were determined. Since we wanted to analyze the transcriptome before differential elongation to observe signal transduction, we captured cells from each side (closer to and further from the nutrient source; **Fig. 1G, H**) of the root tip except the root cap (∼250 µm from quiescent center position) by LMD. These regions lie within the meristem zone according to the general morphology of the main root tips of rice (Li et al. 2015, Jantapo et al. 2021).

**Fig. 2:**
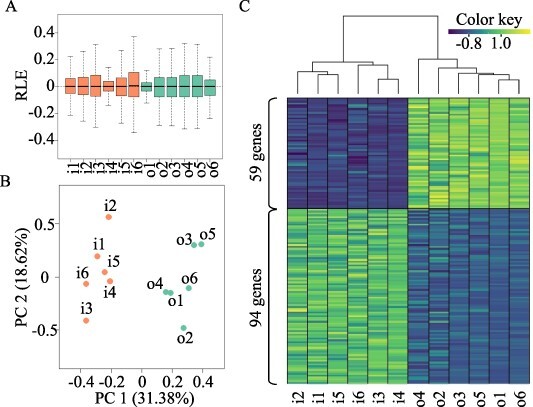
Visualization of the characteristics of gene expression data from RNA sequencing and expression patterns of DEGs between tissues. (A) Box plots and (B) PCA of relative log expression data with normalized counts. Genes differentially expressed between tissues (FDR < 0.05) are defined as DEGs. The results of clustering analysis of the expression patterns of 153 DEGs among the 12 samples are shown in a heatmap of the gene expression levels. In sample names, ‘i’ indicates inside and ‘o’ outside tissues and the numbers indicate replicate.

After RNA-seq analysis, deviations of the relative log expression of all genes were mapped (**Fig. 2A**). As the results of principal component analysis (PCA), PC1 accounted for 31.38% of the total variability in all gene expression data and clearly separated tissue types (**Fig. 2B**). These results imply that our transcriptome data were sufficiently reproducible among replicates and sufficiently distinct between tissues to dissect differences between inside and outside tissues. Discrimination of differentially expressed genes (DEGs) between the two sides identified 94 genes highly expressed (false discovery rate (FDR) < 0.05) in inside tissues and 59 in outside tissues. Hierarchical clustering analysis of the DEGs completely separated the clusters between inside and outside tissues (**Fig. 2C**). Normalized count, fold change and FDR data of all DEGs for 12 samples are summarized in **Supplementary Table S1**.

### GO enrichment analysis

**Fig. 3:**
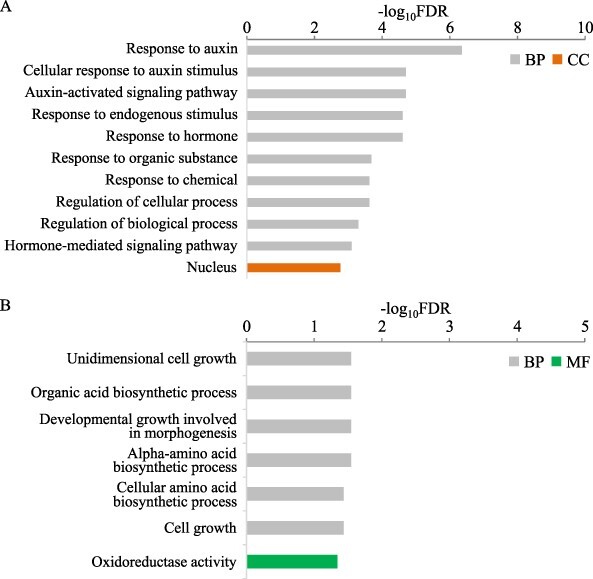
Gene ontology enrichment analysis of 153 differentially expressed genes between inside and outside tissues of root tips in nutritropism. False discovery rates were calculated on the g:Profiler website (https://biit.cs.ut.ee/gprofiler/gost) using DEGs upregulated in the (A) inside and (B) outside tissues. Only the 10 terms enriched with the lowest FDR values are shown for inside tissue. Detailed settings are described in Materials and Methods. BP, biological process; CC, cellular component; MF, molecular function.

In GO enrichment analysis, 55 GO terms (FDR < 0.05) were significantly enriched in DEGs in inside tissues and 7 in outside tissues (**Fig. 3**). Terms including ‘auxin’ were enriched in inside tissue (**Fig. 3A**), but none was found in outside tissue (**Fig. 3B**). This suggests that auxin signaling was activated in the inside tissue in nutritropism. GO terms associated with cell growth and biosynthetic processes were enriched in outside tissue.

### Effects of phytohormone signaling inhibitors on nutritropism

**Fig. 4:**
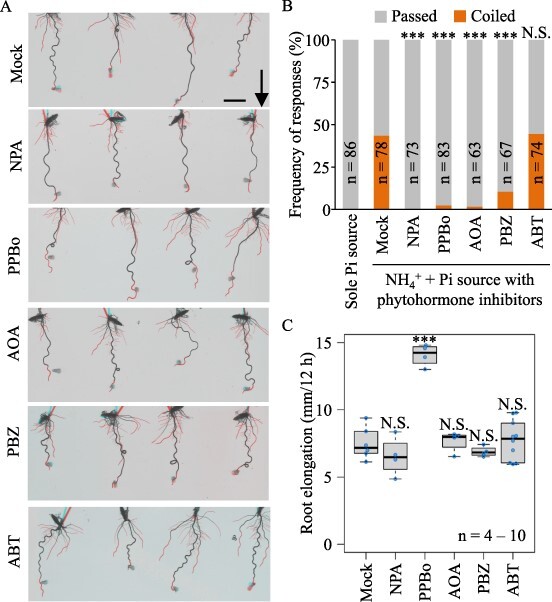
Effects of inhibitors of phytohormone production and signaling on nutritropic responses. Frequencies of passed and coiled responses were determined in the nutritropic bioassay with nutrient sources containing NH_4_^+^ and inorganic phosphate (Pi) at 200 mM with or without the indicated inhibitors (see Materials and methods). Mock sources were prepared without the inhibitors, and Pi alone was prepared as a control without nutritropism. (A) Representative images of the bioassay with indicated inhibitors. Newly grown roots elongated during the 12 h after the insertion of nutrient sources are highlighted in red. Arrow, gravitational direction. Scale bar, 1 cm. (B) Frequencies in the nutritropic bioassay. (C) In the same conditions as (B), elongation during the 12 h after the source insertion was measured for roots passed by the sources. The significance of differences from the mock sources (Mock) was tested using Fisher’s exact test with Holm’s correction for multiple testing in B and Dunnet test in C. N.S., not significant; ***, *P* < 0.001.

The results of our GO enrichment analysis indicated that the nutritropic bending was associated with uneven auxin signaling between inside and outside tissues, so auxin was the most likely candidate for signal transduction. Expression of other phytohormone-related genes also differed between inside and outside tissues, notably genes associated with biosynthesis or signaling of ethylene, gibberellin and salicylic acid (**Table 1**). Therefore, we investigated the relationship of these phytohormones to nutritropism using inhibitors of their transport (NPA for auxin) or biosynthesis (PPBo for auxin, AOA for ethylene, PBZ for gibberellin and ABT for salicylic acid). These inhibitors were added to the nutrient sources to limit the inhibitory effects specifically to the root tips. The effects of inhibitors on nutritropism were evaluated in changes of frequency of nutritropic coiled responses compared with the mock condition as previously described (Yamazaki and Fujiwara 2022). Local exposure of root tips to phytohormone inhibitors did not cause obvious growth inhibition in roots (**Fig. 4A**). The frequency of nutritropic coiled responses was 43.60% (*n* = 78) without inhibitors (mock). However, it was 0.00% (*n* = 73, *P* < 0.001) with NPA, 0.02% (*n* = 83, *P* < 0.001) with PPBo, 1.59% (*n* = 63, *P* < 0.001) with AOA, 10.45% (*n* = 67, *P* < 0.001) with PBZ, and 50.0% (*n* = 64, *P* = 1.00) with ABT (**Fig. 4B**). As another control condition, the frequency in response to a nutrient source without ammonium (sole inorganic phosphate (Pi) source in **Fig. 4B**) was 0.00% (*n* = 86). To prove whether the inhibitions were not caused by their toxic effects on root cell growth, elongation of the tested roots which passed by the nutrient sources with and without the phytohormone inhibitors were compared (**Fig. 4C**). In the elongation during the 12 h in our bioassays, only PPBo treatments significantly changed the root elongation (14.07 mm ± 0.81, *P* < 0.001) compared to the mock condition (7.51 mm ± 1.18). No inhibitor caused inhibition of root elongation in our nutritropic bioassay, and these results indicated that strong inhibitions of nutritropic responses by NPA, PPBo, AOA and PBZ were not caused by inhibiter toxicity to root cells. Taken together, these results showed that nutritropism requires auxin transport and the proper biosynthesis of auxins, ethylene and gibberellins in root tips.

**Table 1:** Differentially expressed genes related to phytohormones. The genes annotated with GO terms including hormones are filtered with a threshold for fold change (>2)

Gene ID	Symbol	Fold change	Related hormone
More expressed in inside tissues			
Os03g0633500	*OsIAA11*	2.53	Auxin
Os04g0608300	*OsSAUR20*	2.77	Auxin
Os03g0727600	*OsACS1*	3.31	Ethylene
Os04g0610400	*OsERF77*	2.28	Ethylene
Os06g0657500	*OsPLT2*	2.16	Ethylene^a^
More expressed in outside tissues			
Os01g0273800	*OsYUCCA9*	2.18	Auxin
Os02g0512000	*OsSAUR10*	3.58	Auxin
Os03g0856700	*OsGA20ox1*	3.17	Gibberellin
Os09g0518200	*OsSGT1*	2.97	Salicylic acid

a (Li and Xue 2011).

### Expression patterns of possible genes downstream of auxin signaling in nutritropism

As described above, it has been accepted that SAURs and expansins promote cell elongations through cell wall modification in shoot phototropism and gravitropism. According to the description of DEGs (**Supplementary Table S1**), one *SAUR* gene, *OsSAUR10* (Os02g0512000), was more expressed in outside tissues. However, two *SAUR* genes, *OsSAUR20* (Os04g0608300) and *OsSAUR22* (Os04g0662200), and two *expansin* genes, *OsEXPA4* (Os05g0477600) and *OsEXPB4* (Os10g0556100), were more expressed in inside tissues, which elongate less than outside tissues in bending roots. We searched for DEGs that include ‘cell wall’ in their GO term to screen for other genes related to cell wall modification (Table 2). Among them, we found three genes annotated with enzymatic activities for cell wall modification: *OsMAN1* (Os01g0663300) and two *expansin* genes, *OsEXPA4* and *OsEXPB4*, were expressed more in inside tissue, and *OsEnS-69* (Os04g0526600) in outside tissue. These genes may play roles in differential cell elongation downstream of auxin signaling.

**Table 2:** Differentially expressed genes annotated with GO terms including ‘Cell wall’

Gene ID	Symbol	Functional description^a^	Fold change
More expressed in inside tissues			
Os01g0663300	*OsMAN1*	similar to (1-4)-β-mannan endohydrolase-like protein	1.69
Os05g0477600	*OsEXPA4*	α-expansin OsEXPA4	1.51
Os08g0420600		Similar to permease 1	1.24
Os10g0556100	*OsEXPB4*	similar to β-expansin EXPB4	1.36
More expressed in outside tissues			
Os02g0532300		α/β hydrolase fold-1 domain-containing protein	2.19
Os02g0735200	*OsGS1;1*	glutamine synthetase 1	1.21
Os04g0526600	*OsEnS-69*	α-amylase/subtilisin inhibitor	2.77
Os04g0613600		40S ribosomal protein S11	3.01
Os05g0403300		peptidase A1 domain-containing protein	1.98
Os09g0518200	*OsSGT1*	UDP: glucose salicylic acid glucosyltransferase	2.97
Os10g0397400	*OsBRD2*	FAD-linked oxidoreductase protein	1.42

a Quoted from monocot PLAZA 5.0.

## Discussion

### Characteristics of our LMD samples and transcriptome data

Continuous nutritropic bending resulted in root coiling around the nutrient source. This phenomenon substantiated all three steps of tropism—namely, perception of external stimuli, signal transduction and differential cell growth. It proved advantageous to obtaining reproducible transcriptome data among replications, as we obtained distinctive transcriptome data from inside and outside tissues of root tips (**Fig. 2**). As far as we know, this is the first report of transcriptome comparison between opposite sides of root tips under tropism. We used a non-reference rice cultivar, WRC25. Because the reference rice cultivar, Nipponbare, also show nutritropism in their lateral roots (Yamazaki et al. 2020), it is supposed that the gene set required for nutritropism is present in the reference genome as well as in WRC25. Therefore, it is worth to analyze the contributions of genes to nutritropism of WRC25 using gene annotations for the reference genome.

### Comparison of auxin distribution between inside and outside tissues

The GO terms ‘Response to auxin’, ‘Cellular response to auxin stimulus’, and ‘Auxin-activated signalling pathway’ were enriched among genes upregulated in inside tissues (**Fig. 3**). This suggests that the ammonium stimulus was transduced into auxin signaling at the more strongly stimulated side of the root tips, and the requirements of auxin signaling were validated by experiments with both transport and biosynthesis inhibitors (**Fig. 4**). Although the actual distribution of auxins between the two sides under nutritropism remains to be elucidated by reporter systems such as DR5 promotor driving β-glucuronidase reporter, higher expression of many *AUX/IAA* genes, *OsIAA3* (Os12g0601400), *OsIAA11* (Os03g0633500), *OsIAA12* (Os03g0633800), *OsIAA15* (Os05g0178600), *OsIAA17* (Os05g0230700), *OsIAA23* (Os06g0597000), and *OsIAA30* (Os12g0601300), strongly suggests that auxins accumulate more in the inside tissues (**Tables 1** and **Supplementary Table S1**). This is because the expression of *AUX/IAA* genes is repressed by AUX/IAA proteins themselves under low auxin levels and upregulated after degradation of the AUX/IAAs via auxin signaling, resulting in a positive correlation with the mRNA levels of *AUX/IAA* genes and auxin levels (Krogan et al. 2014, Li et al. 2016). In gravitropism, an asymmetric auxin distribution is required for gravitational root bending and is established by regulation of auxin transport (Han et al. 2021). The elevated transport of auxins into inside tissues (lower side), promoting differential cell elongation in bending roots, is a well-accepted model. Two *YUCCA* genes responsive to auxin biosynthesis—*OsYUCCA7* (Os04g0128900), *OsYUCCA9* (Os01g0273800)—were included in the nutritropic DEGs (**Supplementary Table S1**), and local application of PPBo, an inhibitor of auxin biosynthesis, strongly and significantly inhibited nutritropic bending, as did NPA, an auxin transport inhibitor (**Fig. 4**). These results suggest that establishment of auxin signaling for nutritropism requires both regulation of auxin transport and *de novo* auxin production in root tips. The requirements of auxin production by *YUCCA* genes for nutritropism or for root bending are further interests.

### Comparison of ethylene and gibberellin distributions between inside and outside tissues

Using DEGs between the both sides as a starting point, we aimed to find the other phytohormone signaling, which are involved in nutritropism. Genes related to the biosynthesis of other phytohormones—*OsACS1* for ethylene (Yamauchi et al. 2015) and *OsGA20ox1* for gibberellin (Kaneko et al. 2003)—were included in DEGs (**Table 1**), and inhibition of *de novo* ethylene and gibberellin production also disrupted nutritropism (**Fig. 4**). These results show that not only auxin but also ethylene and gibberellin signaling orchestrate nutritropism. The distribution of each in root tips under nutritropism is an open question. Although *OsACS1* was highly expressed in inside tissue and *OsGA20ox1* was highly expressed in outside tissue (**Table 1**), mRNA expression levels do not necessarily represent the accumulation of the corresponding phytohormones, as the expression pattern of *OsYUCCA9* is opposite to that of the auxin-responsive *AUX/IAA* repressor genes. We found a known AP2-like ethylene-responsive transcription factor gene, *OsPLT2* (Os06g0657500), whose expression is induced by ethylene treatment (Li and Xue 2011), among the DEGs (**Table 1**). Its higher expression in the inside tissue suggests that ethylene signaling was activated more in the inside tissues in nutritropism.

Based on previous studies, auxin transport required for root gravitropism is enhanced or stabilized by activities downstream of ethylene and gibberellin signaling (Muday et al. 2012, Lofke et al. 2013), so signaling of these three phytohormones also orchestrates gravitropism. Further studies of their interactions in nutritropism will throw light on similarities, differences and interactions between nutritropism and gravitropism.

### Downstream of auxin signaling in nutritropism

In shoot gravitropism, Wang *et al.* found *SAUR* genes upregulated in the lower tissues, corresponding to the outside tissues in our case—that is, elongating tissues (Wang et al. 2020)—and their findings were well consistent with the functional model of SAURs in tropic bending: (1) expression of *SAUR* genes is regulated downstream of auxin signaling, and (2) *SAUR* genes function to acidify the apoplast, which is required for cell wall loosening, by enhancing expansin activities and subsequent cell elongation (Choi et al. 2003, Liscum et al. 2014). Similarly, in shoot gravitropism, expansins are upregulated in elongating tissues, which are auxin-accumulating outside tissues (Esmon et al. 2006). Unlike in shoot gravitropism, however, in root gravitropism the tissues responsible for auxin accumulation and those responsible for elongation differ, and the molecular mechanisms downstream auxin signaling are not established. Therefore, expression patterns of *SAUR* and *expansin* genes in root tips and the functions of these gene families in gravitropism are unknown and interesting. Because our transcriptome data might offer clues about the downstream events, we also filtered *SAUR* and cell-wall modification genes. In our samples, *SAUR* genes *OsSAUR20* (Os04g0608300) and *OsSAUR22* (Os04g0662200) and *expansin* genes *OsEXPA4* (Os05g0477600) and *OsEXPB4* (Os10g0556100) were more expressed in inside tissues (**Tables 1**, **2**, **Supplementary Table S1**), while one *SAUR* gene, *OsSAUR10* (Os02g0512000), and no *expansin* genes were more expressed in outside tissues. Higher expression of these families’ genes in inside tissues is consistent with known downstream events of auxin signaling in shoot gravitropism (Wang et al. 2020) but not with events in elongating tissue. Although some genes of SAUR family may have different functions from acid growth (Stortenbeker and Bemer 2019), previous reports imply that the expression of three *SAUR* genes (*OsSAUR10, OsSAUR20* and *OsSAUR22*) promotes cell elongation (Xu et al. 2022, Huang et al. 2023). Another cell wall modification gene, *OsMAN1*, was also more expressed in inside tissues, and this gene may also promote cell wall loosening in inside tissues of bending roots, because it encodes β-1,4-mannanase, which catalyzes softening and degradation of plant cell walls (Del Carmen Rodríguez-gacio et al. 2012). Although knowledge of genes expressed in outside tissues may also deepen our understanding of differential cell elongation in nutritropism, we found only one gene, *OsEnS-69* (Os04g0526600), annotated with cell wall modification activity (**Table 2**). A barley homologue of *OsEnS-69* functions as a xylanase inhibitor (Sun et al. 2020), and OsEnS-69 may have a role in maintaining cell wall structures rather than in cell wall loosening. Thus, we only detected one *SAUR* gene upregulated in outside tissues, and contributions of the other genes, *SAURs, expansins, OsMAN1* and *OsEnS-69*, to differential cell elongation in nutritropism were totally unexplainable due to the opposite expression patterns to the general expectation except *OsSAUR10*. Therefore, gene functions of cell wall modification activities for nutritropic bending may be controlled by a post-translational regulation rather than transcriptional regulations. It is also possible that there is a requirement of cell-wall loosening in inside tissues rather than outside tissues in the root bending. On the other hand, the GO term ‘Cell growth’ was enriched in the outside tissues (**Fig. 3B**), and *OsGA20ox1, OsBRD2* (Os10g0397400) and *OsILI1* (Os04g0641700) were annotated. *OsGA20ox1 and OsBRD2* are gibberellin and brassinosteroid biosynthesis genes, respectively, and the distribution of the corresponding phytohormones, or their activities promoting cell growth, is not determined from the RNA accumulations as we mentioned above. For *OsILI1*, it was reported that antisense suppression of *OsILI1* expression reduced cell length (Zhang et al. 2009). Thus, higher expression of *OsILI1* in the outside tissues may promote cell elongation, resulting in nutritropic bending.

Although many questions remain about how differential cell elongation is driven after signal transduction in nutritropism as well as gravitropism, our study offers insights into the molecular mechanisms, especially in signal transduction. Although the function of the specific DEGs for nutritropism remains to be experimentally validated, our transcriptome data provide clues to the mechanism of signal transduction and differential elongation in nutritropism and probably interaction with gravitropism in roots.

## Materials and Methods

### Plant materials and growth conditions

We sowed seeds of rice line WRC25, an accession of the World Rice Core Collection (Kojima et al. 2005), provided by the NARO Genebank (https://www.gene.affrc.go.jp/databases-core_collections.php). Sterilized seeds were germinated and grown in square plates (sterile square Schale No. 2, Eiken Chemical Co., Ltd., Tokyo, Japan) held at 60° from the horizontal in a growth chamber under continuous light at 28°C. Each plate contained 60 mL of 1/200-diluted MS medium (pH 5.7–5.8; Murashige and Skoog plant salt mixture, Fujifilm Wako Pure Chemical Corporation, Osaka, Japan) supplemented with 2% (w/v) sucrose (guaranteed reagent; Fujifilm Wako), 0.05% (v/v) Plant Preservative Mixture (Plant Cell Technology Inc., Washington, DC, USA) and 1.5% agar (Agar Purified Powder, Nacalai Tesque, Kyoto, Japan).

### Preparation of nutrient sources to stimulate nutritropic bending

All procedures to prepare the nutrient sources are described in Yamazaki and Fujiwara 2022. Stock solutions of 2 metre NH_4_Cl and NaH_2_PO_4_ · 2H_2_O (Fujifilm Wako) were used as nutrient sources at a final concentration of 200 mM. To the NaH_2_PO_4_ · 2H_2_O stock solution, 1.07 metre NaOH was added to adjust the pH to 7.4. We supplemented the nutrient sources with *N*-1-naphthylphthalamic acid (NPA; Naptalam Standard, Fujifilm Wako) at a final concentration of 200 µM, 4-phenoxyphenylboronic acid (PPBo, Tokyo Chemical Industry Co., Ltd, Tokyo, Japan) at 50 µM, (aminooxy)acetic acid hemihydrochloride (AOA, Fujifilm Wako) at 2 mM, paclobutrazol (PBZ, Tokyo Chemical Industry) at 2 mM or 1-aminobenzotriazole (ABT, Tokyo Chemical Industry) at 2 mM, all prepared first as stock solutions in DMSO. NPA was added as an inhibitor of auxin transport via PINs or ABCBs. PPBo, AOA, PBZ and ABT were added as inhibitors of the biosynthesis of auxins, ethylene, gibberellins and salicylic acid via inhibition of YUCCAs, 1-aminocylopropane-1-carboxylic acid synthase, P450 mono-oxygenase and benzoic acid 2-hydroxylase, respectively. In the mock condition, DMSO was added at 1% (v/v) instead of the stock solutions. All stock solutions were stored at −20°C. Nutrient sources were stored at 4°C and used within 1 month of preparation without inhibitors or 1 week with inhibitors (except that PBZ was used within 1 day).

### Isolation of root tips showing nutritropism and tissue fixation for LMD

The nutrient sources used to generate nutrient gradients were inserted vertically via the tip of a micropipette to reach the bottom of the plate, 3 to 5 mm from the primary or crown root tips of 4- to 7-day-old seedlings in the direction of root elongation (**Fig. 1A**). Root tips that coiled around the sources were defined as exhibiting nutritropism (**Fig. 1B****–****E**), as in our previous paper (Yamazaki and Fujiwara 2022), and coiling root tips observed at 12–24 h after nutrient source insertion were isolated.

### Preparation of paraffin-embedded sections for LMD

The roots were cut and transferred into ice-cold methanol (guaranteed reagent; Fujifilm Wako) for fixation. After 20 min on ice under vacuum, the ice-cold methanol was refreshed. This process was performed twice. The root samples in re-refreshed ice-cold methanol were kept at 4°C until embedding. The methanol was replaced with *n*-butanol (guaranteed reagent; Fujifilm Wako) as follows. After removal of the methanol, ice-cold methanol:*n*-butanol (75:25, v:v) mixture was added and held for 20 min on ice under vacuum. This process was repeated with a methanol:*n*-butanol series (50:50, 25:75, and 3× 0:100, v:v). The *n*-butanol was replaced with melted paraffin wax (Paraplast X-Tra; Fisher Scientific, Pittsburgh, PA, USA) at 60°C in an oven, and the root tips were embedded in the paraffin as described (Takahashi et al. 2010). The samples were then cooled to room temperature and stored at 4°C. For mounting of paraffin-embedded sections, an RNase inhibitor solution—RNAsecure Reagent (Ambion, Austin, TX, USA)—which was diluted 1/25 with RNase-free water and pre-incubated at 60°C, was used on a PEN membrane glass slide (Thermo Fisher Scientific, Waltham, MA, USA). Serial paraffin sections 10 µm thick were prepared on a microtome (RM2135, Leica, Wetzlar, Germany) and were left to float on the surface of the diluted RNase inhibitor solution onto the slides. After incubation at 58°C for 5 min for extension of the sections, the solution was removed with a micropipette and RNase-free paper. The sections were dried at 4°C for 30 min. To remove the paraffin, the slides were gently immersed in Histo-Clear II (National Diagnostics, Atlanta, GA, USA) for 5 min twice, and then were air-dried completely at room temperature.

### LMD

LMD was performed using a Veritas Laser Microdissection System LCC1704 (Molecular Devices, Sunnyvale, CA, USA) as described (Takahashi et al. 2010). The root tip area within ∼250 µm of the quiescent center was collected. Cells located on the inside and outside of the curving root tips were collected separately from every section (**Fig. 1F****–****H**). Tissues from several root tips were mixed for a single biological replicate of RNA sequencing, and six biological replicates for each side were analyzed.

### Extraction and quantification of total RNA

From cells collected by LMD, total RNA was extracted with a PicoPure RNA Isolation Kit (Thermo Fisher Scientific). RNA concentrations were determined with Quant-iT RiboGreen RNA reagents (Invitrogen, Carlsbad, CA, USA). With an RNA 6000 Pico Kit on an Agilent 2100 Bioanalyzer (Agilent Technologies, Santa Clara, CA, USA), RNA quality was estimated by the RNA Integrity Number (RIN) calculated in 2100 Expert software (Agilent, v. B.02.02, eukaryote total RNA pico mode) as described (Takahashi et al. 2010). RNA concentrations, yields and RIN scores of all samples are shown in **Supplementary Table S2**.

### RNA-seq analysis

RNA-seq libraries were prepared as described (Koembuoy et al. 2020). cDNA for short-read sequencing was synthesized from the extracted RNA with a SMART-Seq v4 Ultra Low Input RNA Kit (Clontech Laboratories Inc., Mountain View, CA, USA). Sequencing libraries were constructed with a Nextera XT DNA Library Preparation Kit (Illumina Inc., San Diego, CA, USA) using double-stranded cDNA with 12 cycles of PCR amplification. Then, paired-end 100-bp sequencing was performed on the Illumina HiSeq 2500 platform. We obtained >10 million reads per sample, with a mean value of 12.7 million reads. Library sizes were normalized for preparation of the gene expression data. Gene expression patterns among samples were examined by PCA.

All raw reads were quality controlled and filtered in fastp software (Chen et al. 2018). Clean data of high quality (phred quality ≥ 15) and >15 bp in length were used for RNA-seq analysis. All clean reads were mapped in HAISAT2 software (Kim et al. 2019) to the IRGSP-1.0 Nipponbare reference genome in RAP-DB (https://rapdb.dna.affrc.go.jp) (Kawahara et al. 2013, Sakai et al. 2013). Mapped reads of each gene were counted by featureCounts (Liao et al. 2014) using Oryza_sativa.IRGSP-1.0.54.gtf from EnsemblPlants (ftp://ftp.ensemblgenomes.org/pub/release-54) as a gene annotation file. All of the raw sequencing data and sequencing datasets are available in DDBJ (PRJDB16535).

### Identification and analysis of differentially expressed genes

To obtain DEGs, we performed normalization and differential expression analysis in the RUVSeq (Risso et al. 2014). The parameter *k* was set as 6 to reduce ‘unwanted variation’ effects among biological replications. The default settings for the other parameters were used. DEGs between inside and outside tissues were statistically determined by using a FDR < 0.05 as the cutoff value and were used for later analysis. GO enrichment in DEGs was analyzed in the g:GOSt tool in g:Profiler (https://biit.cs.ut.ee/gprofiler/gost) (Raudvere et al. 2019) with organism set as ‘Oryza sativa Japonica Group’, statistical domain scope as ‘Only annotated genes’, significance threshold as ‘Benjamini–Hochberg FDR < 0.05’ and default settings for the others.

## Supplementary Material

pcae003_Supp

## Data Availability

All data supporting the findings of this study are available within the paper and within its supplementary data published online. RNA-seq data have been deposited in the NCBI BioProject database under accession number PRJDB16535.
